# Dynamics of Heat Shock Protein 70 Serum Levels As a Predictor of Clinical Response in Non-Small-Cell Lung Cancer and Correlation with the Hypoxia-Related Marker Osteopontin

**DOI:** 10.3389/fimmu.2017.01305

**Published:** 2017-10-18

**Authors:** Christian Ostheimer, Sophie Gunther, Matthias Bache, Dirk Vordermark, Gabriele Multhoff

**Affiliations:** ^1^Department of Radiation Oncology, Martin Luther University Halle-Wittenberg, Halle, Germany; ^2^Department of Radiation Oncology, Klinikum rechts der Isar, Technische Universität München (TUM), Munich, Germany

**Keywords:** heat shock protein 70, osteopontin, radio(chemo)therapy, non-small-cell lung cancer, therapy response, overall survival

## Abstract

Hypoxia mediates resistance to radio(chemo)therapy (RT) by stimulating the synthesis of hypoxia-related genes, such as osteopontin (OPN) and stress proteins, including the major stress-inducible heat shock protein 70 (Hsp70). Apart from its intracellular localization, Hsp70 is also present on the plasma membrane of viable tumor cells that actively release it in lipid vesicles with biophysical characteristics of exosomes. Exosomal Hsp70 contributes to radioresistance while Hsp70 derived from dying tumor cells can serve as a stimulator of immune cells. Given these opposing traits of extracellular Hsp70 and the unsatisfactory outcome of locally advanced lung tumors, we investigated the role of Hsp70 in the plasma of patients with advanced, non-metastasized non-small-cell lung cancer (NSCLC) before (T1) and 4–6 weeks after RT (T2) in relation to OPN as potential biomarkers for clinical response. Plasma levels of Hsp70 correlate with those of OPN at T1, and high OPN levels are significantly associated with a decreased overall survival (OS). Due to a therapy-induced reduction in viable tumor mass after RT Hsp70 plasma levels dropped significantly at T2 (*p* = 0.016). However, with respect to the immunostimulatory capacity of Hsp70 derived from dying tumor cells, patients with higher post-therapeutic Hsp70 levels showed a significantly better response to RT (*p* = 0.034) than those with lower levels at T2. In summary, high OPN plasma levels at T1 are indicative for poor OS, whereas elevated post-therapeutic Hsp70 plasma levels together with a drop of Hsp70 between T1 and T2, successfully predict favorable responses to RT. Monitoring the dynamics of Hsp70 in NSCLC patients before and after RT can provide additional predictive information for clinical outcome and therefore might allow a more rapid therapy adaptation.

## Introduction

Lung cancer is the second most common tumor type in the Western world that accounts for the majority of cancer-related deaths worldwide (1, 2). The lack of specific symptoms limits the possibilities to diagnose lung cancer at an early stage when radical surgery or stereotactic ablative radiotherapy can assure long-term tumor control and cure (3). As a result, most patients are diagnosed in advanced tumor stages where curative-intended treatment options are limited (4, 5). Cure of locally advanced (inoperable) NSCLC after definitive RT (4, 5), as the gold standard, have failed to improve survival significantly (6). Immune-based therapies are in the scientific focus (7) for their remarkable clinical responses that have been reported for some tumor types (8–10). Particularly the treatment with immune checkpoint inhibitors such as nivolumab (11–16) caused a paradigm shift in the therapy of NSCLC. Despite promising remission rates, overall survival (OS) still remains dismal at only 10–20% in almost all patients. This emphasizes the medical need for integration of immune-oncologic approaches into other treatment concepts that are based on an improved patient stratification (17, 18).

Evidence is accumulating that apart from direct cytotoxic effects, RT can elicit systemic antitumor immune responses (19, 20) by modulating the tumor and its microenvironment (21–24). However, the patient’s individual immune competence and immune escape mechanisms often hamper radio(chemo)therapy (RT)-induced abscopal effects (25). These findings further accentuate the necessity of a pretreatment patient stratification (26, 27), and a continuous monitoring of immune responses during therapy (28). Presently, circulating proteins, exosomes, microRNAs and immune cell subpopulations are discussed as potential prognostic and predictive markers (29–33). We studied the dynamics of extracellular heat shock protein 70 (Hsp70) levels and correlated it with osteopontin (OPN) plasma levels at diagnosis (T1) as predictors for outcome. Elevated levels of these biomarkers are generally associated with an aggressive tumor phenotype (34, 35). OPN is reported to be associated with reduced intratumoral pO_2_ levels, which is prognostic for NSCLC patients (36–38). In a randomized double-blinded trial, elevated OPN plasma levels were able to identify patients with head and neck cancer who showed clinical benefit from a hypoxia sensitizer after RT (39). For NSCLC patients, an additive prognostic value after radical RT could be determined for the co-detection of hypoxia- and angiogenesis-related markers OPN, vascular endothelial growth factor (VEGF), and carbonic anhydrase IX (CAIX) (30). Furthermore, the serial detection of circulating OPN plasma levels provided additional prognostic information for NSCLC patients in stage III with respect to the risk to relapse (31).

Heat shock protein 70 fulfils different tasks, depending on its associated partners and its sub- or extracellular localization. Vaccination with Hsp70-peptide complexes isolated from tumor cells can elicit CD8^+^ T cell specific antitumor immune responses (40, 41), membrane-bound Hsp70 serves as a tumor-specific target for Hsp70-activated natural killer (NK) cells (42–45), and circulating Hsp70 can act as a biomarker for monitoring outcome in patients with head and neck cancer (46). Extracellular Hsp70 can originate from two major sources, exosomal Hsp70 which is actively released by viable, membrane Hsp70-positive tumor cells and free Hsp70 which most likely originates from dying cells (47, 48). By using lipHsp70 enzyme-linked immunosorbent assay (ELISA), it is possible to detect both forms of Hsp70 quantitatively in serum and plasma (49). Recent data of our group demonstrated a significant correlation of Hsp70 levels and vital tumor mass, but also provided evidence that free extracellular Hsp70 in a pro-inflammatory cytokine milieu can activate innate immunity in NSCLC patients (50).

In this prospective clinical trial, we evaluated the predictive quality of circulating pre- and post-therapeutic Hsp70 and OPN levels at T1 in patients with non-metastasized advanced NSCLC.

## Patients and Methods

### Patients and Treatment

A total of 44 patients with advanced NSCLC (M0) were prospectively recruited into a clinical study at the hospital of Martin Luther University Halle-Wittenberg. The inclusion criteria were (i) age ≥ 18 years, (ii) histologically confirmed, nonoperable NSCLC, (iii) no prior treatment, and (iv) indication for RT, as determined by the interdisciplinary tumor board. The Ethics Committee of the Medical Faculty of the Martin Luther University Halle-Wittenberg approved the study protocol. Written informed consent was obtained from all patients before start of the study. All procedures were in accordance with the Helsinki Declaration of 1975 (as revised in 2008). Staging was based on the TNM classification of malignant tumors (7th edition) and treatment was carried out at the Department of Radiation Oncology of the Medical Faculty of the Martin Luther University Halle-Wittenberg. Depending on the World Health Organization performance status and comorbidities, treatment consisted of a three-dimensional conformal, normofractionated (5 Fx/week) definite RT (single dose 2 Gy, total dose 66 Gy, Siemens Primus, Germany) ± double-agent based chemotherapy (cisplatin 20 mg/m^2^ body surface, day 1–5 and 29–33; vinorelbine 25 mg/m^2^ body surface, used on day 1 and 29) in treatment week one and five (2 courses). RT was computed tomography-based (GE Healthcare) and all patients received a FDG positron emission tomography-scan prior to RT which was used for target volume delineation (Oncentra Masterplan External Beam software, Nucletron, Elekta, USA). The first follow-up of the patients was performed 4–6 weeks after end of RT to evaluate their post-radiotherapeutic response at the Department of Radiation Oncology, University Hospital Halle-Wittenberg. Thereafter, patients were followed up regularly every 3 months for a period of 5 years according to RT guidelines. The mean follow-up period in patients alive was 34 (22–48) months. The survival status of patients was continuously monitored in cooperation with local citizen registration offices.

A positive therapy response was defined as complete remission, implying a disappearance of all target lesions, or partial remission which commonly signifies a decrease of at least 30% in the lesion with the largest diameter. A negative therapy response is defined by stable or progressive disease.

### Plasma Samples

Blood samples of NSCLC patients were collected by peripheral venous puncture before start of RT (T1) and 4–6 weeks after the end of RT (T2). Briefly, blood was collected in two S-Monovette EDTA KA/9 ml tubes (Sarstedt, Nümbrecht, Germany). Blood was anti-coagulated and centrifuged at 4°C for 10 min with 4,000 rpm. Aliquots of 150–300 µl were prepared and directly stored at −80°C for further analysis.

### Detection of Hsp70 and OPN

Heat shock protein 70 concentrations were determined using the lipHsp70 ELISA, which is capable to detect both, lipid-bound and free Hsp70 in serum and plasma. The use of cmHsp70.1 as detection antibody (49, 51) allows quantitative analysis of total content of circulating Hsp70 in the blood. 96-well MaxiSorp Nunc-Immuno plates (Thermo, Rochester, NY, USA) were coated overnight with 2 µg/ml rabbit polyclonal antibody (Davids, Biotechnologie, Regensburg, Germany) directed against human Hsp70 in sodium carbonate buffer (0.1 M sodium carbonate, 0.1 M sodium hydrogen carbonate, pH 9.6). After three washing steps with phosphate buffered saline (PBS, Life Technologies, Carlsbad, CA, USA) with 0.05% Tween 20 (Calbiochem, Merck, Darmstadt, Germany), wells were blocked with 2% milk powder (Carl Roth, Karlsruhe, Germany) in PBS for 1.5 h at 27°C. After another washing step, samples diluted 1:5 in CrossDown Buffer (Applichem, Chicago, IL, USA) were added to the wells for 2 h at 27°C. After another washing, wells were incubated with 4 µg/ml of the biotinylated mouse-anti-human monoclonal antibody cmHsp70.1 (multimmune, Munich, Germany) in 2% milk powder in PBS for 2 h at 27°C. After a last washing step, 0.2 µg/ml horseradish peroxidase-conjugated streptavidin (Pierce, Thermo, Rockford, IL, USA) in 1% bovine serum albumin (Sigma-Aldrich, St. Louis, MO, USA) was added for 1 h at 27°C. Binding was quantified by adding substrate reagent (R&D Systems, Minneapolis, MN, USA) for 30 min at 27°C and absorbance was read at 450 nm, corrected by absorbance at 570 nm, in a Microplate Reader (BioTek, Winooski, VT, USA). Each sample was measured in duplicates in three independent experiments. An eight-point standard curve using recombinant Hsp70 diluted in CrossDown Buffer at concentrations ranging from 0 to 50 ng/ml, as well as reference plasma samples were used as internal controls for each individual assay.

For OPN, the “Human Osteopontin Assay” ELISA (IBL Ltd., Japan) was performed and optical density was measured blinded and in duplicate according to manufacturer’s instructions. To determine the OPN concentration, the standard curve supplied by the kit was used and OPN plasma concentration is reported in ng/ml (±1 SD). None of the two markers shows an age- and/or gender-related association.

### Statistical Analysis

All statistical analyses were performed using the SPSS PASW software package for windows (SPSS Inc., USA, version 19.0) and statistical significance was accepted with two-sided *p*-values (*p* < 0.05). Median Hsp70 plasma levels were used as cutoff values.

Non-parametric tests (Mann–Whitney *U* test, Kruskal–Wallis *H* test) were used to determine statistically significant differences in patient subgroups with low and high Hsp70 concentration with and without response. Differences in Hsp70 levels in patients with and without therapy response were investigated using Pearson’s chi-squared test. Coherences between Hsp70 and OPN as a hypoxia-related marker were evaluated using Pearson’s rank correlation coefficient and paired samples test assessed potential differences in plasma Hsp70 levels before and after RT. Survival analysis was performed using the Kaplan–Meier product limit method with the log-rank test. The survival status of the patients was monitored and determined with the help of local citizen registration offices. Overall survival (OS) was calculated from start of radiotherapy until death or last seen in follow-up.

Therapy response was the primary endpoint, classified in responding (complete or partial remission after RT) vs. non-responding patients (progressive or stable disease after RT). For univariate and multivariate analysis, the Cox proportional hazard regression model was used to calculate the relative risk and hazard ratio and its 95% confidence interval (CI). Receiver operating characteristic (ROC) curves illustrate the performance of Hsp70 plasma levels as a binary classifier system in the prediction of therapy response after RT.

## Results

### Pre-Therapeutic (T1) OPN Levels Correlate with Hsp70 Plasma Levels in Patients with NSCLC

A total of 44 NSCLC patients (6 females, 38 males) with NSCLC (M0) were enrolled into the study for T1. The clinico-pathological characteristics of all patients (*n* = 44) are summarized in Table 1 and that of non-responding and responding patients is shown in Table 2. With respect to tumor volume (*p* = 0.086), age (*p* = 0.114), gender (*p* = 0.306), histology (*p* = 0.158), and UICC stage (*p* = 0.175), no statistically significant differences have been determined in non-responding and responding patients.

**Table 1:** Clinico-pathological characteristics of all non-small-cell lung cancer patients (*n* = 44) at M0 (Martin Luther University Hospital, Halle-Wittenberg).

		Counts	
Gender	Female	6	14%
	Male	38	86%
Histological type	Squamous cell carcinoma	23	52%
	Adeno ca	19	43%
	Other	2	5%
UICC stage	I–II	2	5%
	IIIa	16	36%
	IIIb	26	59%

**Table 2:** Clinico-pathological characteristics of non-responding and responding non-small-cell lung cancer patients at M0 (Martin Luther University Hospital, Halle-Wittenberg).

		Non-responder		Responder	
		
		Counts		Counts	
Gender	Female	0		6	17.6%
	Male	10	100%	28	82.4%
Histological type	Squamous cell carcinoma	7	70%	16	47%
	Adeno ca	2	20%	17	50%
	Other	1	10%	1	3%
UICC stage	I–II	1	10%	1	2.9%
	IIIa	4	40%	12	35.3%
	IIIb	5	50%	21	61.8%

**Figure 1:**
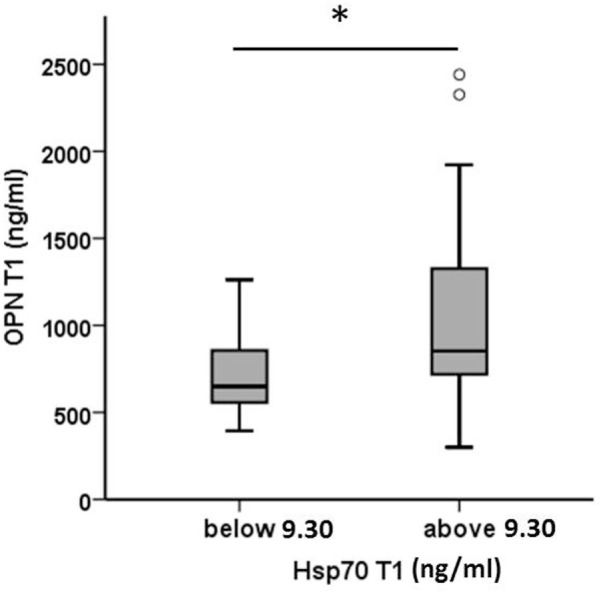
Comparison of pre-therapeutic (T1) osteopontin (OPN) levels in non-small-cell lung cancer patients (M0) with high and low median heat shock protein 70 (Hsp70) plasma levels. According to the median Hsp70 plasma level of 9.30 ng/ml the patient cohort (*n* = 43) was divided into two subgroups with median Hsp70 plasma levels below or above 9.30 ng/ml; Mann–Whitney *U* test, *p* = 0.021.

Plasma levels of OPN and Hsp70 were determined pre-therapeutically (T1, before RT) in all patients (Table 3). As already shown for a larger patient cohort (30), also in a subgroup of non-metastasized NSCLC patients, high pre-therapeutic OPN plasma levels (above median, *n* = 22) significantly correlated with inferior OS compared to low (below median, *n* = 22) levels (13 [5–66] vs. 23 [5–61]; *p* < 0.05). Both biomarkers, OPN and Hsp70, revealed a positive correlation according to the Pearson’s correlation coefficient (*r* = 0.422, *p* = 0.005) for T1. According to the Mann–Whitney *U* test (Figure 1), patients whose median Hsp70 values were above 9.30 ng/ml showed significantly higher OPN values, compared to those with median Hsp70 values below 9.30 ng/ml (*n* = 43, *p* = 0.021). A direct comparison of the OPN and Hsp70 values also revealed a correlation (*r* = 0.42), as shown in the Supplementary material (Figure S1 in Supplementary Material).

**Table 3:** Comparison of pre-therapeutic (T1) osteopontin (OPN) and heat shock protein 70 (Hsp70) plasma levels in non-small-cell lung cancer patients (M0; *n* = 44) in relation to overall survival.

	OPN T1 (ng/ml)	Hsp70 T1 (ng/ml)
*N* (missing)	44 (0)	43 (1)
Mean	872.14	12.13
SEM	71.63	2.02
Median	752.45	9.30
SD	475.11	13.26
Maximum	2441.00	67.50
Minimum	299.30	0.20
Paired samples test (overall survival)	*p* < 0.05	

### Hsp70 Plasma Levels Drop after RT in Patients with NSCLC

To investigate the impact of RT on Hsp70 plasma levels, pre- (T1) and post- (T2) therapeutic Hsp70 plasma levels were compared in 26 patients from whom plasma samples were available at both time-points. As summarized in Table 4, median Hsp70 levels dropped significantly from 10.35 before RT to 6.05 ng/ml after RT (paired samples test, *p* = 0.016). According to the Pearson’s correlation coefficient, a significant positive correlation was determined (*r* = 0.659, *p* < 0.0001).

**Table 4:** Comparison of heat shock protein 70 (Hsp70) plasma levels before (T1) and 4–6 weeks after (T2) radio(chemo)therapy in non-small-cell lung cancer patients (M0).

	Hsp70 T1 (ng/ml)	Hsp70 T2 (ng/ml)
*N*	26	26
Mean	14.94	9.02
SEM	3.02	1.81
Median	10.35	6.05
SD	15.41	9.24
Maximum	67.50	46.20
Minimum	0.20	0.80
Paired samples test	*p* = 0.016	

The drop in circulating Hsp70 after RT was also detected by mean Hsp70 levels (T1 vs. T2: 14.94 vs. 9.02 ng/ml). However, compared to a cohort of 114 healthy donors (7.8 ng/ml) which was published previously (49), mean Hsp70 values in NSCLC patients remained to be significantly upregulated before (T1) and after (T2) RT (*p* < 0.05).

### High Post-Therapeutic Hsp70 Plasma Levels Predict Clinical Response to RT

To address the question whether Hsp70 might be predictive for clinical response, pre- (T1) and post- (T2) therapeutic Hsp70 plasma levels were associated with response to RT. As expected, patients who responded to therapy showed a significantly improved OS compared to non-responding patients (23 vs. 9 months, *p* = 0.026, log-rank Mantel Cox) who had an increased risk of death (*r* = 2.11, CI [0.94–4.57], *p* = 0.58).

**Figure 2:**
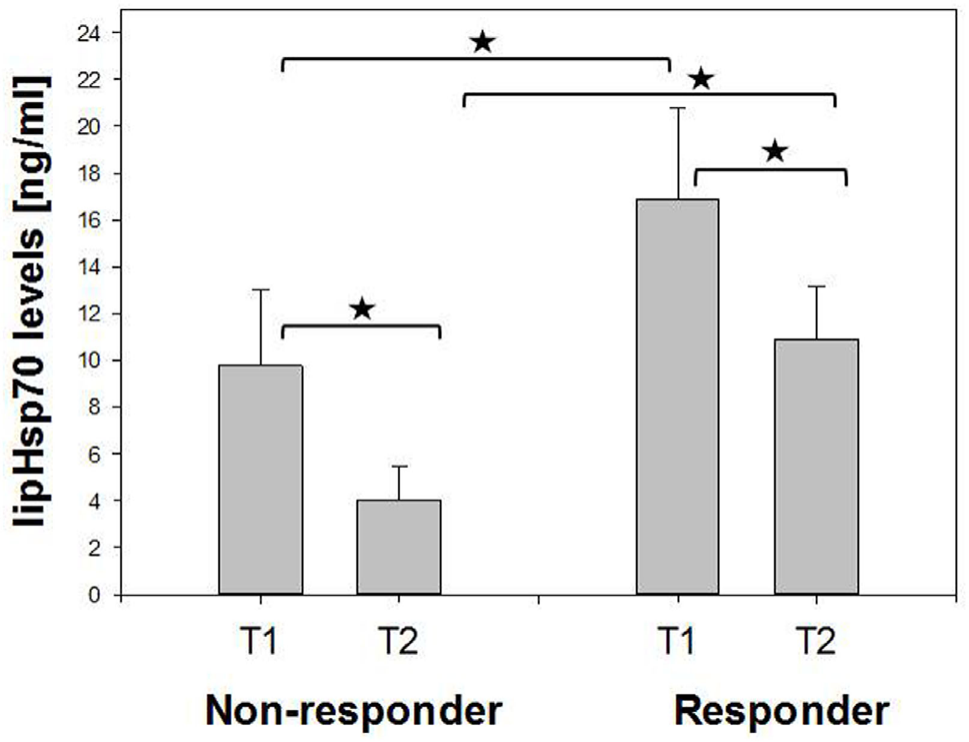
Comparison of mean pre- (T1) and post- (T2) therapeutic heat shock protein 70 (Hsp70) plasma levels in non-responding and responding non-small-cell lung cancer patients (M0). Non-responder: 7, responder: 19. Mann–Whitney *U* test, *p < 0.05.

A comparison of Hsp70 plasma levels before (T1) and 4–6 weeks after RT (T2) in non-responding (*n* = 7, 9.76 vs. 4.03 ng/ml) and responding patients (*n* = 19, 16.85 vs. 10.87 ng/ml) demonstrated that in both patient subgroups, mean and median Hsp70 plasma levels declined after RT (Figure 2). In general, responding patients had significantly higher (mean/median) Hsp70 plasma levels compared to non-responding patients at T1 and T2 (Figure 2).

**Figure 3:**
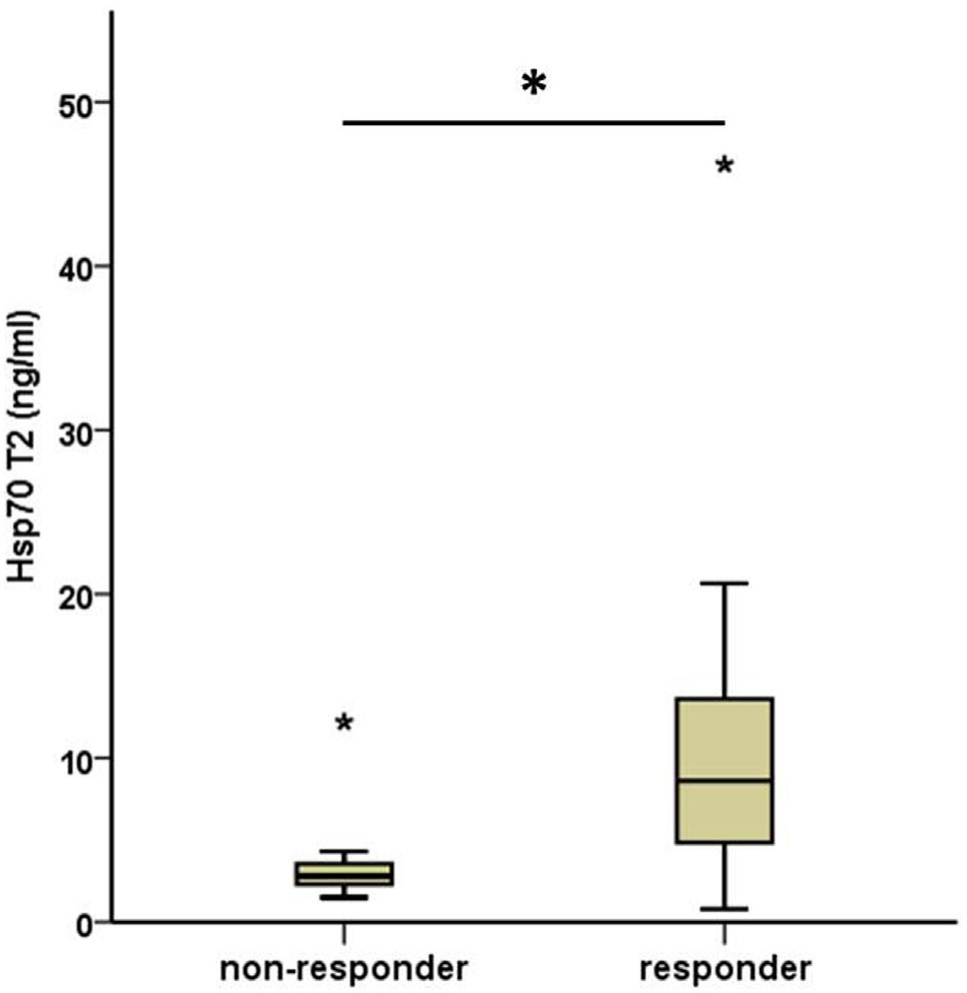
Comparison of median post- (T2) therapeutic heat shock protein 70 (Hsp70) plasma levels in non-responding and responding non-small-cell lung cancer patients (M0). Non-responder: 7, responder: 19. Mann–Whitney *U* test, asterisks above the box plots indicate outliers; *p = 0.013.

As depicted in Figure 3, all patients who responded to therapy had significantly higher median Hsp70 plasma levels at T2 (median 8.6 ng/ml, range 0.8–46.2) after RT compared to those who showed no response (median 2.8, range 1.5–12.2) 4–6 weeks after therapy (T2) (Mann–Whitney *U* test, *p* = 0.013). The median Hsp70 values, revealed similar results with respect to both time-points in responding (11.10 ng/ml at T1 vs. 8.60 ng/ml at T2) and non-responding (5.30 ng/ml at T1 vs. 2.80 ng/ml at T2) patients (paired samples test *p* = 0.034).

**Figure 4:**
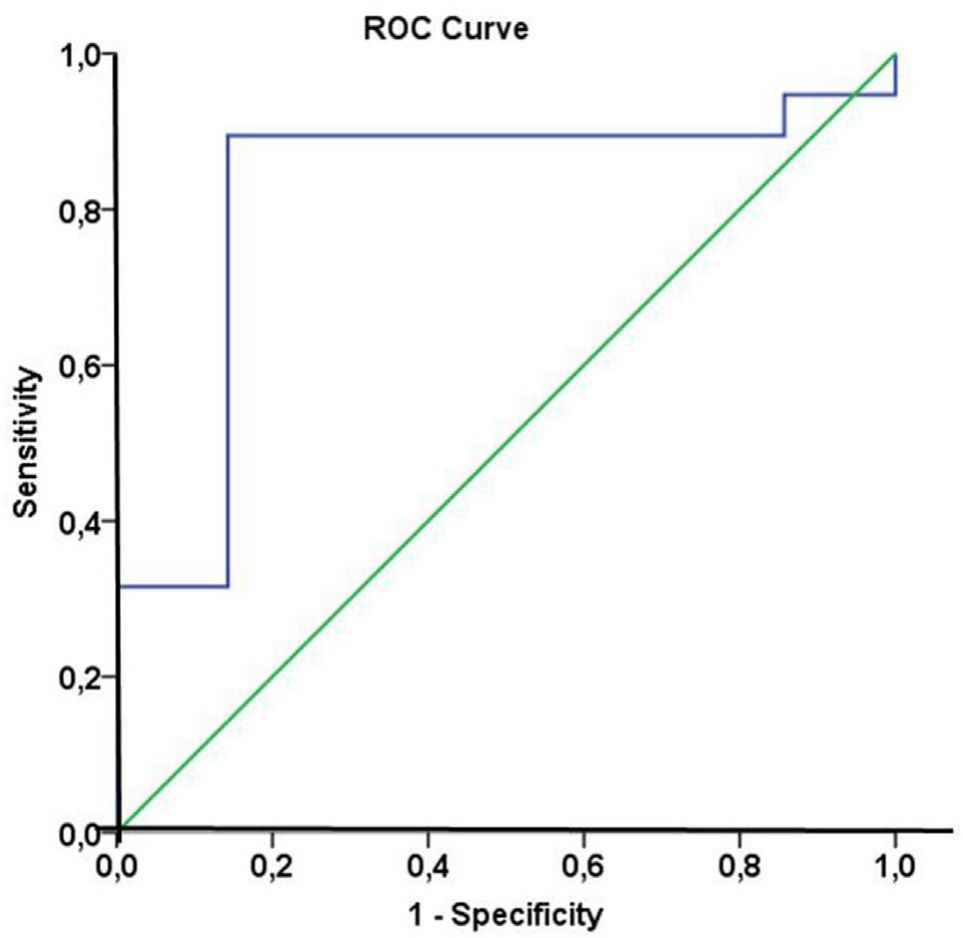
Receiver operating characteristic (ROC) curve analysis of heat shock protein 70 (Hsp70) plasma levels of non-small-cell lung cancer (NSCLC) patients (M0) to predict therapy response. Area under the curve = 0.82, *p* = 0.014. The optimal cutoff value for distinction of responders and non-responders was an Hsp70 plasma level of 4.35 ng/ml with a sensitivity of 0.895 and a false positive rate of 0.143.

In line with these findings, a subdivision of the patient cohort into subgroups with median Hsp70 plasma levels above and below 5.0 ng/ml at T2 revealed that patients with Hsp70 plasma levels above the threshold had significantly higher response rates than those below the threshold (92.9 vs. 50%, Pearson Chi-Square *p* = 0.02) (Table 5). Based on these findings, plasma Hsp70 levels were analyzed for their potential to predict therapy response after RT. The related ROC curve analysis (Figure 4) showed a significant predictive function (*p* = 0.014) of plasma Hsp70 levels for therapy response with an area under the curve (AUC) of 0.82. The optimal cutoff value which determines a positive therapy response is a value of ≤4.35 ng/ml with a sensitivity of 0.895 and a false positive rate of 0.143. Plasma Hsp70 levels which were taken before start of therapy (T1) showed a similar trend, but failed statistical significance.

**Table 5:** Comparison of post- (T2) therapeutic heat shock protein 70 (Hsp70) plasma levels in non-responding and responding non-small-cell lung cancer patients (M0).

Therapy response (T2)	Median Hsp70 above 5 ng/ml	Median Hsp70 below 5 ng/ml
*N*	14	12
Non-responder	1 (7%)	6 (50%)
Responder	13 (93%)	6 (50%)
Pearson chi-square	*p* = 0.02	

*Patients were divided into two subgroups with high and low median Hsp70 levels*.

## Discussion

### Post-Therapeutic Hsp70 Plasma Levels As a Biomarker for Therapy Response

The patient’s endogenous immune defense is able to attack tumor cells. However, tumors as well as its microenvironment have developed mechanisms that allow immune escape against tumor cells (52–55). Therefore, novel therapeutic approaches aim to reactivate the patient’s immune defense by inhibiting tumor-induced immune checkpoints that impair immune responses against malignantly transformed cells (56, 57). Another strategy to reinforce the patient’s immune system is based on the activation of immune effector cells against tumor-specific targets that are overexpressed in tumor cells, presented on the cell surface and released in a tumor-selective manner (58). Tumor cells of different types including NSCLC (34) frequently overexpress Hsp70, present it on their plasma membrane, and actively release it in tumor exosomes (46, 58–60). However, dying tumor cells also release Hsp70, most likely as a free protein (47, 48). High intracellular Hsp70 levels have been found to interfere with apoptotic pathways and thereby protect tumor cells from programmed cell death following stress (61). Hence, we assume that therapy-resistant tumor cells with high cytosolic Hsp70 levels can better compensate RT-induced damage and thus mediate tumor cell survival.

Murakami et al. showed that in addition to cytosolic also membrane-bound Hsp70 supports protection of tumor cells against RT-induced cell death (62), although the expression density of cytosolic and membrane-bound Hsp70 are not associated. Therefore, membrane Hsp70 fulfils dual roles, on the one hand, it acts as a tumor-specific target for Hsp70-activated NK cells (43, 44, 50, 63), on the other hand it mediates therapy resistance. An ongoing phase II clinical trial using *ex vivo* Hsp70-activated NK cells for the treatment of patients with NSCLC after RT is presently testing whether the immunostimulatory capacity of NK cells can overrule therapy resistance of membrane Hsp70-positive NSCLC (44).

In the present trial, we investigated the role of circulating Hsp70 as a prognostic marker to predict outcome of RT in patients with NSCLC (M0) at different time-points. A comparison of pre- and post-therapeutic plasma levels revealed significantly elevated Hsp70 levels in responding compared to non-responding patients.

The lipHsp70 ELISA (52) detects both, lipid-bound and free Hsp70. We hypothesize that high Hsp70 levels at diagnosis predominantly originate from exosomal Hsp70 released by viable tumor cells. This is in line with our finding that Hsp70 plasma levels before start of therapy reflect vital gross tumor volume (50). In contrast, elevated post-therapeutic Hsp70 plasma levels rather originate from dying tumor cells (47, 48) that might be able to stimulate the immune system. Analysis of the concentration of cytosolic proteins in the exosomal versus non-exosomal plasma fraction after ultracentrifugation of responding non-metastasized NSCLC patients in stage IIIa/b (*N* = 4) before (T1) and after RT (T2) showed a significant (*p* < 0.05) protein drop in the exosomal fraction and an increase in the non-exosomal fraction after therapy (data not shown) which reflects the reduction in viable tumor mass after therapy. In summary, pre- and post-treatment Hsp70 levels are indicative for different tumor characteristics such as vital tumor mass, intrinsic tumor aggressiveness, and RT-induced tumor cell death that can cause immunostimulation.

Previous work of our group demonstrated that membrane Hsp70 serves as a target for NK cells that have been pre-stimulated with an Hsp70-peptide plus low-dose interleukin 2 (42). The stimulation of NK cells is associated with an upregulated expression of activatory NK receptors, including the C-type lectin receptor CD94/NKG2C (64) that in turn induces the production of the pro-apoptotic enzyme granzyme B (63). With respect to these findings, we speculate that high post-therapeutic Hsp70 plasma levels derived from dying tumor cells in a pro-inflammotory environment after RT might be able to stimulate Hsp70-reactive NK cells that mediate favorable therapeutic outcome. Elevated pre-therapeutic exosomal Hsp70 plasma levels might be predictive for a superior outcome because they reflect an aggressive, but yet immunogenic tumor type. This is in accordance with the finding that Hsp70-bearing exosomes isolated from membrane Hsp70-positive tumor cells, but not from their Hsp70-negative counterparts, can induce the migratory and cytolytic activity of NK cells (65).

The high predictive value of post-therapeutic Hsp70 plasma levels for the response of an individual patient could be demonstrated by ROC analysis with an AUC of 0.82 (*p* = 0.014). Identical analyses have been performed for Hsp70 plasma levels before start of therapy. Although a similar trend was observed, pre-therapeutic levels failed to show statistical significance as a biomarker for clinical response. This might be explained by the fact that pre-therapeutic Hsp70 plasma levels predominantly originate from viable tumor cells and thus represent vital tumor mass rather than therapy response.

To obtain a better view on the exact dynamics and prognostic relevance of circulating Hsp70 levels, further pre- and post-therapeutic measurements of Hsp70 at different time-points have to be correlated with clinical response in larger patient cohorts.

### Role of Hsp70 in the Context of Hypoxia-Related Markers of the Tumor Microenvironment

Another parameter that co-determines OS of patients after RT is the tumor microenvironment. The presence of hypoxic stress impacts prognosis and therapy response to RT adversely. The molecular effects induced by RT involve the production of DNA radicals which are normally fixed by oxygen. Hence, DNA damage decreased under hypoxic stress and hypoxia-inducible factor 1α (HIF1α) is stabilized which in turn leads to promotion of tumor cell survival. HIF1α also has been shown to impair membrane Hsp70 expression on tumor cells, and therefore might negatively affect NK cell recognition (66).

Tumor hypoxia is also associated with an overexpression of OPN. Physiologically, OPN is involved in the process of bone remodeling (67); however, many tumor cells show an overexpression of this protein (68) as an aggression marker. Plasma levels of OPN correlate with tumor hypoxia in NSCLC (38), and previously we demonstrated that high OPN levels before RT and increasing OPN levels after RT translate into poor OS in NSCLC after radical RT (30, 31). With respect to the prediction of therapy response, OPN as a single marker failed to show significance. Only in combination with VEGF and CAIX, the prognostic impact of OPN could be augmented (30). Hypoxic stress also has been shown to increase the release of exosomes (69) that contain large amounts Hsp70. Therefore, the present study evaluated the prognostic and predictive value of Hsp70 levels in relation to OPN. Due to a positive correlation of Hsp70 and OPN plasma levels at diagnosis, the association of OPN and OS was re-evaluated in the subgroup of non-metastasized NSCLC patients (*n* = 44) that was also analyzed for Hsp70. In line with previous results of a larger, more heterogenous NSCLC patient cohort (31), non-metastastized NSCLC patients also revealed a significant correlation of high OPN values at T1 with a decreased OS.

## Conclusion

Our findings illustrate the differential prognostic and predictive relevance of pre- and post-treatment Hsp70 levels in NSCLC patients after RT. Being actively released by viable tumor cells in exosomes, high pre-therapeutic Hsp70 levels (T1) are most likely be indicative for viable tumor mass. Therapy response that was associated with a reduction in tumor size results in a significant drop in exosomal Hsp70 plasma levels from 21.7 ± 2.8 to 15.6 ± 1.3 ng/ml (*p* < 0.05, data not shown), as determined by lipHsp70 ELISA in 4 responding patients who were not included into the trial. However, with respect to the immunostimulatory capacity of Hsp70 derived from dying tumor cells, elevated post-therapeutic Hsp70 levels also can predict beneficial outcome to RT. Although elevated OPN levels significantly correlate with decreased OS, reduced lung function, and weight loss (30), OPN as a single parameter is unable to predict therapy response (30, 31). Therefore, the co-detection of both biomarkers before and/or after RT integrates prognostic (OPN) and predictive (Hsp70) information for therapy response that allows a more rapid therapy adaptation to improve clinical outcome of NSCLC patients.

## Ethics Statement

The Ethics Committee of the Medical Faculty of the Martin Luther University Halle-Wittenberg approved the study protocol. Written informed consent was obtained from all patients before start of the study. All procedures were in accordance with the Helsinki Declaration of 1975 (as revised in 2008).

## Author Contributions

CO and SG equally contributed to the study; CO, SG, and MB conceived and designed the experiments, analyzed data, and wrote the paper; DV gave clinical advice; and GM did proof-reading, designed and revised the study.

## Conflict of Interest Statement

The authors declare that the research was conducted in the absence of any commercial or financial relationships that could be construed as a potential conflict of interest.

## Abbreviations

AUC, area under the curve; CAIX, carbonic anhydrase IX; CD, cluster of differentiation; CT, computed tomography; ELISA, enzyme-linked immunosorbent assay; Hsp70, heat shock protein 70; HIF1α, hypoxia-inducible factor 1α; IL-2, interleukin 2; NK cells, natural killer cells; NSCLC, non-small-cell lung cancer; OPN, osteopontin, PAI-1, plasminogen activator inhibitor type 1; PBS, phosphate buffered saline; PET, positron emission tomography; RT, radio(chemo)therapy; ROC curve, receiver operating characteristic; SCC, squamous cell carcinoma; uPAR, urokinase type plasminogen activator receptor; WHO, World Health Organization; VEGF, vascular endothelial growth factor.
