# Supplementary figures and images for: Dynamics of Heat Shock Protein 70 Serum Levels As a Predictor of Clinical Response in Non-Small-Cell Lung Cancer and Correlation with the Hypoxia-Related Marker Osteopontin
Source: Front Immunol. 2017 Oct 18;8:1305. doi: 10.3389/fimmu.2017.01305 (PMC5651249; doi:10.3389/fimmu.2017.01305)

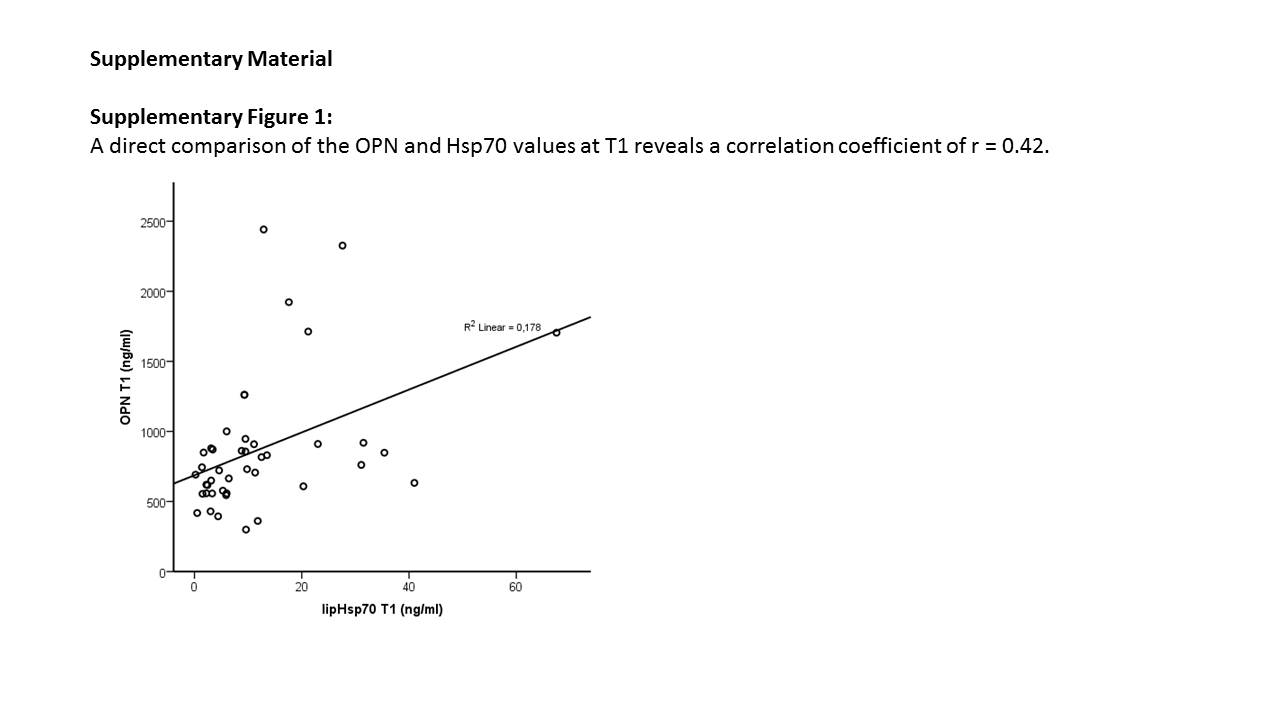

Supplement: Supplementary file 1 [file image_1.jpeg]

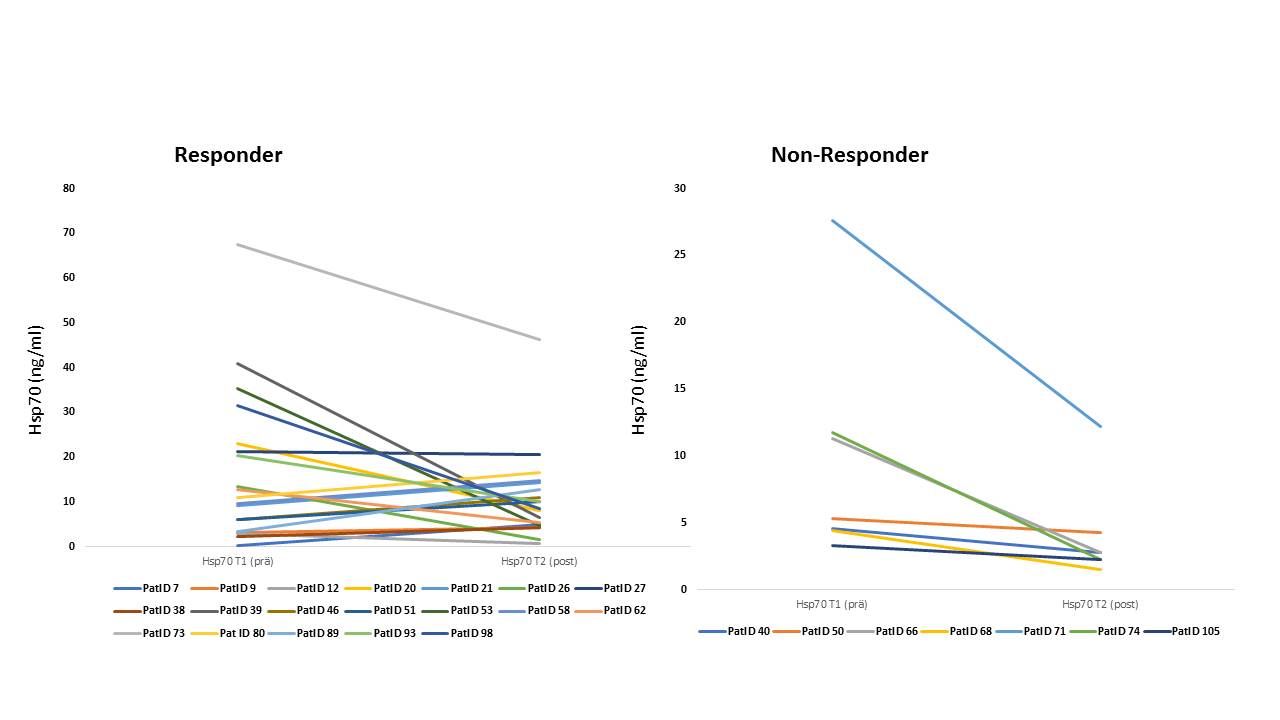

Supplement: Supplementary file 2 [file image_2.jpeg]
